# Liquid Biopsy for Early Pancreatic Cancer Detection: Why Has It Not Yet Worked?

**DOI:** 10.3390/cancers18030525

**Published:** 2026-02-05

**Authors:** Kenji Takahashi, Yusuke Ono, Kenzui Taniue, Krushna C. Patra, Takuya Yamamoto, Mikihiro Fujiya, Yusuke Mizukami

**Affiliations:** 1 Division of Gastroenterology, Department of Internal Medicine, Asahikawa Medical University, Asahikawa 078-8510, Hokkaido, Japan; 2 Center for Intractable Diseases and ImmunoGenomics, National Institutes of Biomedical Innovation, Health and Nutrition, Osaka 567-0085, Japan; 3 Institute of Biomedical Research, Sapporo Higashi Tokushukai Hospital, Sapporo 065-0033, Hokkaido, Japan; 4 Isotope Science Center, The University of Tokyo, Bunkyo-ku, Tokyo 113-0032, Japan; 5 Department of Cancer Biology, University of Cincinnati, Cincinnati, OH 45267, USA

**Keywords:** liquid biopsy, cell-free DNA (cfDNA), cell-free RNA (cfRNA), extracellular vesicles (EVs), non-coding RNA (ncRNA), pancreatic cancer (PDA)

## Abstract

Despite extensive technological advances and an ever-growing body of literature, liquid biopsy has yet to achieve reliable early detection of pancreatic ductal adenocarcinoma (PDA). Numerous studies have investigated circulating tumor-derived components, including cell-free DNA (cfDNA), cell-free RNA (cfRNA), extracellular vesicles (EVs), and circulating tumor cells (CTCs), primarily using peripheral blood samples; however, their clinical utility for early-stage disease remains limited. The fundamental obstacles are biological rather than purely technical: early PDA and its precursor lesions, such as pancreatic intraepithelial neoplasia (PanIN) and intraductal papillary mucinous neoplasms (IPMN), are characterized by minimal tumor burden, low levels of nucleic acid shedding, and substantial background signals from non-neoplastic tissues. Increasing analytical complexity through multilayered liquid biopsy approaches, including analyses from pancreas-associated fluid, has not consistently translated into improved diagnostic performance and, in some cases, has amplified issues related to specificity, reproducibility, and interpretability. Moreover, molecular alterations detected in body fluids may reflect clonal expansion without inevitable malignant progression, raising concerns regarding overdiagnosis and clinical decision-making. Pre-analytical variability, lack of standardization, and limited access to tumor-adjacent fluids further hinder clinical implementation. Liquid biopsy should therefore be regarded as a complementary modality rather than a substitute for histopathological diagnosis, with its precise clinical role in early detection still ill-defined. In this review, we critically examine why liquid biopsy has not yet succeeded in early PDA detection, highlighting the key biological, technical, and clinical barriers that must be addressed to move the field beyond exploratory research toward meaningful clinical application.

## Simple Summary

Liquid biopsy is anticipated as a non-invasive complement to histopathology, yet its utility for early detection of pancreatic ductal adenocarcinoma (PDA) remains limited. The primary obstacles are biological—specifically minimal tumor shedding—rather than purely technical, hindering clinical implementation. We critically examine these barriers and the lack of standardization. Furthermore, we outline potential solutions and initiatives to overcome these challenges, incorporating our own clinical attempts and experimental data to bridge the gap toward meaningful clinical application.

## 1. Introduction

Pancreatic ductal adenocarcinoma (PDA) is characterized by aggressive invasion and metastasis, and the most dismal cancer. Due to this aggressive nature, surgical intervention is often limited to a minority of cases [1,2]. PDA remains a formidable challenge in oncology due to its high lethality. Although localized, potentially curable disease is found in fewer than 25% of cases, the overall 5-year survival rate stands at 6–8% [3]. A primary contributor to this poor prognosis is the difficulty of early-stage diagnosis. While diagnostic strategies utilizing imaging and body fluids have evolved over the last decade, limitations persist. Carbohydrate antigen 19-9 (CA19-9) is currently the standard and most sensitive biomarker; however, it demonstrates suboptimal performance, with a sensitivity of 70–80% and specificity below 50%. Notably, its sensitivity drops to 55.6% for Stage I PDAC, indicating its inadequacy for early detection [4]. Consequently, there is an urgent need for reliable biomarkers to facilitate the diagnosis of early-stage PDA. The term ‘early PDA’ refers to Stage 0 (carcinoma in situ) and Stage I according to the UICC classification in this review.

**Figure 1:**
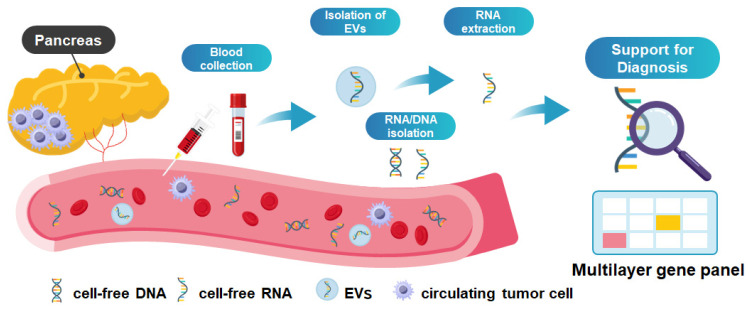
Overview of Liquid Biopsy for generating multilayer gene panel.

Recently, extensive research has been conducted on liquid biopsy as a minimally invasive testing strategy using various body fluid samples, including blood, urine, and saliva, with the aim of enabling early cancer diagnosis. A wide range of tumor-derived targets in body fluids have been investigated for liquid biopsy, such as cell-free DNA (cfDNA), cell-free RNA (cfRNA), circulating tumor cells (CTCs), extracellular vesicles (EVs), and proteins [5,6,7] (Figure 1). In the field of pancreatobiliary diseases, gastrointestinal biofluids, duodenal fluid (DF) and pancreatic juice (PJ) collected during endoscopic procedure, can be analyzed in addition to blood or urine [8].

Despite substantial research efforts and a growing number of published studies, only a limited number of liquid biopsy approaches have progressed toward clinical implementation, and biomarkers that clearly outperform the conventional marker CA19-9 have not yet been established [4]. The underlying reasons are multifactorial and include challenges in achieving sufficient diagnostic accuracy and reproducibility, limited sensitivity for detecting early-stage disease, and pronounced tumor heterogeneity. To address these issues, diverse strategies are currently being explored across multiple disciplines, including advances in analytical technologies, assay design, and specimen selection [2,9,10].

In this review, we focus on the fundamental challenges in biomarker development for the early diagnosis of PDA. We also summarize the current landscape of recent liquid biopsy research and ongoing efforts to overcome these limitations, with a particular emphasis on cfDNA, cfRNA, and EV-based approaches, including our investigative attempts. This review used a search strategy using PubMed with the terms ‘Liquid biopsy’ and ‘Pancreatic cancer’ focusing on the literature published since 2020.

## 2. Current Landscape of Liquid Biopsy in PDA

### 2.1. Overview of Biomarkers and the Diagnostic Gold Standard

The main liquid biopsy biomarkers currently being studied include ctDNA, CTCs, and EVs, such as exosomes. Regarding the performance of these markers, each has different strengths and weaknesses. ctDNA is useful for finding specific mutations like KRAS. However, Bettegowda et al. reported that early-stage PDA releases much less DNA into the blood compared to other tumors [11]. Therefore, the sensitivity of ctDNA is often low in Stage 0 or I patients. On the other hand, CTCs provide whole-cell information, which helps us understand the tumor’s biological character. But these cells are extremely rare in patients without metastasis, making them difficult to use for screening [12,13]. In contrast, EVs are highly stable and abundant in the circulation. They carry rich information, such as proteins and RNAs. Recent studies suggest that EV analysis can distinguish early cancer from benign diseases with high accuracy [14]. While these markers are promising, the main problem is the small number of tumor-derived materials in the early stages. Overcoming this ‘biological barrier’ is the most important challenge for clinical application in early diagnosis [6].

Currently, Endoscopic Ultrasound-guided Tissue Acquisition (EUS-TA) represents the state-of-the-art standard for the definitive pathological diagnosis of solid pancreatic lesions and serves as the reference against which liquid biopsy must be compared. The field has evolved from standard Fine-Needle Aspiration (FNA) toward Fine-Needle Biopsy (FNB) to obtain better histological cores. Recent comprehensive meta-analyses have confirmed that improved EUS-TA techniques, particularly FNB, offer superior diagnostic accuracy and histological yield compared to conventional methods [15,16]. Despite high diagnostic accuracy for visible masses, the invasive nature of EUS remains a limitation for repeated sampling or screening purposes. Therefore, liquid biopsy would be considered to have an important position to complement endoscopic tissue biopsy.

### 2.2. Biological and Anatomical Barriers Specific to PDA

Liquid biopsy in PDA faces distinct anatomical and biological hurdles compared to other solid tumors. Unlike lung cancer, where biomarkers enter systemic circulation directly, PDA biomarkers drain into the portal vein and pass through the liver first [17]. This “first-pass” hepatic filtration removes a significant amount of tumor DNA before it reaches the peripheral blood. This issue is compounded by the dense tissue structure (desmoplasia) and low number of blood vessels in pancreatic tumors, which physically trap tumor DNA and prevent it from shedding into the blood [18]. Furthermore, inflammation from conditions like chronic pancreatitis creates biological “noise,” making it harder to distinguish cancer from benign disease without reducing test sensitivity [19]. These factors combine to suppress circulating tumor signals, often keeping them below the detection limit of standard assays.

### 2.3. Quantitative Evidence of Diagnostic Sensitivity in Early Stages

Multiple high-impact, large-scale studies provide compelling quantitative evidence substantiating the significant biological barriers in early PDA detection via liquid biopsy. A seminal study by Bettegowda et al. established a direct correlation between tumor burden and ctDNA detectability, reporting mutation detection in only 48% of localized (Stage I/II) PDA compared to over 75% in metastatic PDA [11]. This stage-dependent sensitivity was further corroborated by landmark multi-cancer detection studies. The CancerSEEK study demonstrated that while maintaining high specificity (>99%) against healthy controls, sensitivity for PDA dropped drastically to approximately 40% in Stage I, compared to around 80% in Stage III [20]. Similarly, the large-scale CCGA study utilizing methylation signatures observed consistent trends, confirming limited sensitivity for Stages I and II across methodologies [21]. Taken together, these robust empirical data quantitatively underscore the formidable challenge of reliably detecting early PDA due to insufficient shedding of biomarkers from small, localized tumors.

## 3. Liquid Biopsy Using ctDNA: Current Limitations and Strategies to Address Them

### 3.1. Genetic Landscape and Emerging Fragmentomic Markers

The development of pancreatic cancer precursor lesions, such as pancreatic intraepithelial neoplasia (PanIN) and intraductal papillary mucinous neoplasms (IPMN), is frequently associated with activating mutations in driver genes including *KRAS* and *GNAS* [22]. Subsequent inactivation of tumor suppressor genes, including *SMAD4*, *TP53*, and *CDKN2A*, promotes progression of these precancerous lesions to invasive pancreatic ductal adenocarcinoma (PDA) [22]. Accordingly, DNA mutation analysis using cfDNA has been regarded as one of the most central targets in liquid biopsy-based approaches for PDA diagnosis [23].

Beyond somatic mutations, numerous PDA-specific DNA methylation markers have been identified, and their combined application has been proposed as a promising strategy for early diagnosis [24,25,26,27]. In addition, methylation-based approaches such as methylated CpG tandem amplification and sequencing (MCTA-Seq) have generated multiple candidate biomarkers with high diagnostic accuracy [28,29]. Furthermore, recent studies have demonstrated that cfDNA fragmentation patterns, including fragment sizes and end motifs, differ between patients with PDA and healthy individuals. Machine learning models integrating these fragmentomic features have been suggested to contribute to early diagnosis and, potentially, cell-of-origin inference [30]. Nevertheless, despite the clinical implementation of plasma cfDNA analysis for cancer genome profiling (CGP) tests in Japan, its application remains limited to therapeutic decision-making rather than primary cancer diagnosis [31].

### 3.2. Technical Challenges: Sensitivity, Stability, and Biological Noise

Plasma cfDNA-based liquid biopsy offers several advantages, including minimal invasiveness, ease of sample collection enabling longitudinal monitoring, short turnaround time (TAT), and the ability to capture systemic tumor heterogeneity rather than information from a single lesion. However, cfDNA concentrations in peripheral blood are inherently low, highly fragmented, and susceptible to chemical degradation. Moreover, the proportion of tumor-derived ctDNA within total cfDNA is strongly influenced by tumor size, stage, and the proximity to circulating body fluids, resulting in limited mutation detection rates in early PDA [32].

Pre-analytical factors further complicate cfDNA analysis. Delays between sample collection and processing, as well as variations in storage conditions, can substantially affect the analytical results. For example, leukocyte lysis during blood storage releases genomic DNA, increasing background DNA levels and reducing variant allele frequency (VAF), thereby impairing the detection of low-frequency mutations [33]. Standardized protocols for plasma separation, storage, and processing are therefore essential. Additionally, mutations arising from clonal hematopoiesis of indeterminate potential (CHIP), which increases with aging, represent a major source of false-positive findings. Since hematopoietic cells constitute the primary source of cfDNA, CHIP-associated mutations, including those in *DNMT3A* and *TET2*, as well as PDA-relevant genes such as *TP53* and *BRCA1/2* may be misidentified as tumor-derived alterations [34,35], thereby reducing diagnostic specificity.

### 3.3. Analytical Methodologies and Future Strategies

Genetic analysis of cfDNA can be broadly classified into PCR amplification-based detection methods and sequencing-based genomic profiling approaches that enable broader genomic coverage. PCR-based techniques include real-time PCR, digital PCR (dPCR), and the extremely sensitive BEAMing method, which is particularly suited for detecting predefined hotspot mutations. These approaches offer high analytical sensitivity (approximately 0.01% for dPCR and BEAMing), relatively low cost, and simplified workflows; however, they are inherently limited in the number of target regions that can be interrogated simultaneously.

In contrast, sequencing-based cfDNA analysis incorporating molecular barcoding and error-suppression strategies allow for the detection of low-frequency variants at allele frequencies of approximately 0.1–1%, which is substantially lower than those detectable by standard target sequencing approaches without error correction. Compared with PCR-based assays, sequencing-based genomic profiling enables the simultaneous identification of a broader range of genomic alterations, including point mutations and copy number variations across multiple genes, thereby providing a more comprehensive overview of tumor-associated genomic changes [5,36].

**Figure 2:**
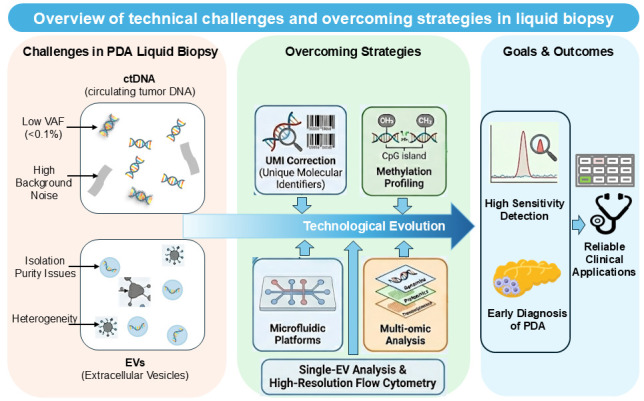
Overview of technical challenges and overcoming strategies in liquid biopsy.

To overcome these limitations, various strategies are currently being explored. These include the use of pre-amplification combined with dPCR to enhance sensitivity for low-abundance mutations, as well as a multiplexed assay design developed by our group to maximize information yield from limited sample volumes [37]. In parallel, specialized blood collection tubes capable of stabilizing nucleic acids (e.g., Streck, Becton Dickinson, and Roche) have been introduced, with expanding applications for both cfDNA and cfRNA preservation (Figure 2).

## 4. Liquid Biopsy Using EV and RNA: Complementary Approaches and Remaining Challenges

### 4.1. Diagnostic Potential of Non-Coding RNAs and Extracellular Vesicles

As discussed in the previous section, cfDNA-based detection of *KRAS* mutations has intrinsic limitations, particularly in early-stage disease. To complement its diagnostic performance, EVs and non-coding RNAs (ncRNAs) have emerged as alternative and potentially informative targets in liquid biopsy research.

ncRNAs are broadly classified based on transcript length into small ncRNAs (<200 nucleotides) including microRNAs (miRNAs), and long non-coding RNAs (lncRNAs) exceeding of 200 nucleotides [38,39]. Among these, miRNAs, typically 21–25 nucleotides in length, have been most extensively investigated as circulating biomarkers [40,41], whereas studies on lncRNAs remain relatively limited [39,42,43]. Several reports have demonstrated the diagnostic potential of miRNAs detected as cfRNA in body fluids. For example, Nakamura et al. validated a 13-miRNA signature for PDA detection, archiving an area under the curve (AUC) of 0.93 [44]. Another study employing machine learning of urinary miRNA combinations reported high diagnostic accuracy for PDA, including early-stage disease [45].

**Figure 3:**
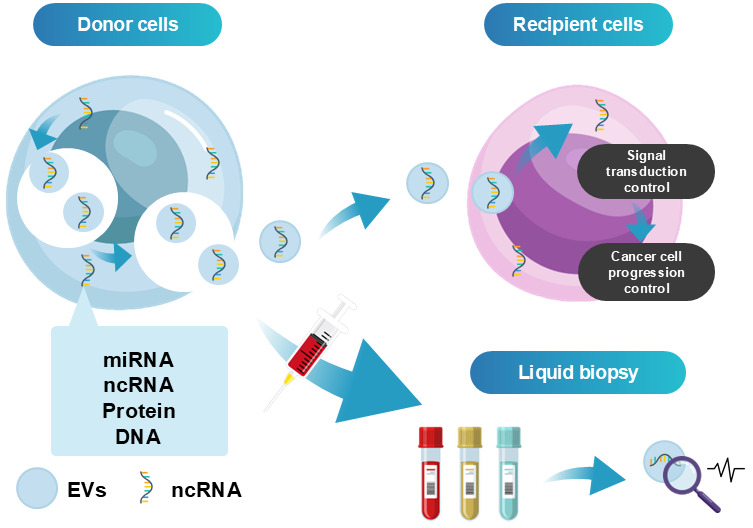
Clinical application of EV-targeted liquid biopsy.

EVs are membrane-bound vesicles secreted by virtually all cell types and are present in diverse body fluids, including blood, urine, breast milk, and saliva. EVs contain a variety of bioactive molecules, such as proteins and nucleic acids, reflecting the molecular characteristics of their cells of origin [46,47]. Recent studies have demonstrated the diagnostic potential of EV-associated markers in pancreatic diseases (Figure 3). For instance, elevated expression of MUC5AC in plasma EVs was shown to distinguish high-grade IPMNs from low-grade IPMNs with high sensitivity and specificity [48].

### 4.2. Overcoming Isolation Hurdles: Novel Strategies and Biomarker Discovery

Despite their potential, EV-based biomarker development faces substantial technical challenges, particularly regarding isolation methods, purity, and reproducibility. Commonly used EV isolation techniques, such as precipitation and ultracentrifugation, are prone to co-isolation of non-EV contaminants and inter-method variability [46]. To address these issues, we have developed a proprietary EV isolation platform, EViSTEP^®^, based on immunoprecipitation using antibody-coupled magnetic particles targeting the tetraspanin, combined with a pretreatment reagent that enhances EV–antibody interactions while reducing contaminating proteins [49]. Using this platform, we identified a previously unrecognized lncRNA, HEVEPA, which was significantly upregulated in serum EVs from PDA patients compared to healthy controls and IPMN patients, achieving an AUC of 0.86. Notably, HEVEPA elevation was observed specifically within EVs rather than as cfRNA, underscoring the value of EV-targeted biomarker discovery [49] (Figure 2).

### 4.3. The Unresolved Issue of Standardization

Despite these advances, standardization remains a major challenge in cfRNA and EV analyses, particularly with respect to endogenous reference controls. While several stable miRNAs (e.g., miR-149-3p, miR-2861, and miR-4463) have been proposed as reference for cfRNA [50], no consensus has been established for EV-based normalization strategies. In our preliminary analysis, ACTB and B2M demonstrated relatively stable expression within EVs, and further validation studies are ongoing.

## 5. Recent Advances in Liquid Biopsy: Research Trends and Clinical Translation

### 5.1. Emerging Protein Biomarkers and Alternative Biofluids

In recent years, several promising biomarkers have been introduced into clinical practice under health insurance coverage in Japan. Among these, the APOA2 isoform index (APO2-iTQ) has been implemented in Japan as an adjunctive marker for PDA diagnosis. Multiple studies have demonstrated significantly reduced APOA2-ATQ/AT levels in patients with PDA, with reported AUC values comparable to or exceeding those of CA19-9 in distinguishing early disease stages [51,52,53,54]. While established in Japan, widespread international adoption will require further validation in diverse ethnic cohorts to satisfy regulatory criteria globally.

Another promising marker is EphA2-NF, a circulating N-terminal fragment of the receptor tyrosine kinase EphA2, which is frequently overexpressed in PDA cells. EphA2-NF is independent of CA19-9 and has been associated with poor prognosis among patients receiving gemcitabine plus nab-paclitaxel (GnP) treatment, suggesting its potential clinical utility as both a diagnostic and prognostic marker [55].

Beyond blood-based markers, tumor-adjacent fluids have also attracted attention as alternative liquid biopsy sources. Yachida et al. reported high diagnostic accuracy for PDA using *KRAS* mutation analysis of DF collected after secretin [56], while analysis of S100P protein expression in DF has shown potential utility as a minimally invasive screening approach [57,58]. Furthermore, as summarized in Table 1, numerous additional candidates—ranging from EV-associated miRNAs to circular RNAs (circRNAs) and lncRNAs—have been identified. Several of these novel markers have demonstrated diagnostic performance comparable to or exceeding that of CA19-9, offering a diverse arsenal of potential tools for early detection [59,60,61,62,63,64].

### 5.2. Technological Innovations: Single-Cell Analysis and Activatable Probes

To overcome the biological barriers described in previous sections, novel technological approaches are being developed. The integration of single-cell technologies represents a pivotal advancement in liquid biopsy, moving beyond simple enumeration to high-resolution molecular characterization. Single-cell RNA sequencing (scRNA-seq) has been instrumental in dissecting the intratumoral heterogeneity of PDA, which often limits the sensitivity of bulk assays. For instance, Franses et al. utilized scRNA-seq on CTCs to identify a distinct ‘basal-ZEB1+’ phenotype enriched in epithelial–mesenchymal transition markers, demonstrating that this specific subpopulation significantly correlates with poor prognosis and therapeutic resistance [13]. Similarly, Raghavan et al. revealed how the tumor microenvironment drives cell state plasticity between basal-like and classical phenotypes. Their analysis of the tumor secretome based on single-cell transcriptomics provides a roadmap for identifying novel, subtype-specific secreted proteins or surface markers detectable in blood [65]. Furthermore, to address low sensitivity and background noise, Fu et al. introduced biomarker-activatable molecular probes. By utilizing a specific ‘turn-on’ mechanism triggered by the tumor microenvironment, these probes significantly enhance the signal-to-noise ratio, enabling precise early stratification and real-time therapeutic monitoring [66].

### 5.3. Paradigm Shift Toward Multi-Omics and Clinical Implementation

Ultimately, integrating multi-omics datasets is considered a critical step toward improving the performance of early diagnosis. Recent studies have demonstrated that integrating multiple biomarker modalities significantly enhances diagnostic efficacy compared to single-analyte assays. For instance, the landmark ‘CancerSEEK’ study employed a multi-analyte approach combining ctDNA mutations with circulating protein markers, successfully increasing sensitivity for early-stage cancers without compromising specificity [20]. Similarly, AI-driven models integrating cfDNA fragmentomics with genomic alterations have achieved high diagnostic performance, with AUC values approaching 0.99 in some comparative studies [30,67].

Currently, the focus of clinical translation is moving toward ‘Multi-Cancer Early Detection (MCED).’ For example, the PATHFINDER study tested this approach in more than 6000 participants. It successfully detected a cancer signal in about 1.4% of the people, and diagnostic workups confirmed cancer in a significant number of these cases [68]. Based on these results, the NHS-Galleri trial (ISRCTN91431511) has started in the UK, enrolling 140,000 asymptomatic participants to evaluate whether methylation-based screening can decrease the number of late-stage cancers, including PDA [69]. Collectively, these studies underscore the extensive global efforts directed toward biomarker discovery. As observed in lung cancer where ctDNA analysis guides standard care [5,70], the potential of liquid biopsy for PDA is evident. However, rather than relying on a single molecular target, integrating multiple biomarkers across different platforms offers a more reliable path toward improved diagnostic accuracy (Figure 2). Consistent with this approach, we are currently developing a multi-layered diagnostic platform centered on driver gene profiling combined with cfRNA and EV RNA analysis. These multimodal strategies substantiate that fusing distinct biological signals—such as DNA, RNA, or proteins—through advanced computational modeling is a pivotal strategy to overcome the biological barriers of low tumor shedding and high background noise. Accordingly, we propose the development and clinical implementation of a cost-effective ‘Pancreatic Cancer Diagnostic Panel’ that combines complementary biomarkers, as conceptually illustrated in Figure 1.

## 6. Conclusions

### Future Perspectives

Despite substantial advances in liquid biopsy technologies, early detection of PDA remains an unresolved clinical challenge. This could be primarily due to biological constraints, such as the minimal abundance of tumor-derived nucleic acids and other factors, marked tumor heterogeneity, and the difficulty in discriminating indolent precursor lesions from those with malignant potential. Increasing analytical sophistication has not been sufficient to overcome these limitations yet. Also, the transition from research to clinical practice faces biological as well as significant translational barriers. These include the lack of standardized protocols for sample processing, the prohibitive costs associated with high-throughput sequencing, and the stringent requirements for regulatory approval. To bridge these gaps, systematic approaches are imperative. First, the development of industry-wide standardization and consensus guidelines would be essential to ensure reproducibility across institutions. Second, large-scale prospective cohort studies are required to validate clinical utility and justify regulatory approval. Finally, rigorous cost-effectiveness analyses are necessary to facilitate healthcare policy adaptation and insurance reimbursement.

Consequently, liquid biopsy would be realistically positioned not as a standalone screening tool, but as a complementary modality that supports pathological diagnosis and risk stratification in well-defined clinical settings. Future progress will depend on aligning technological innovation with biological insight and clinical feasibility. By integrating multilayer-modal panels with robust clinical evidence, liquid biopsy can achieve meaningful clinical implementation in pancreatic cancer care.

## Figures and Tables

**Table 1:** Recent findings in liquid biopsy research for PDA.

Analysis Target	Molecular Target	Type of Clinical Sample	Potential Roles for Liquid Biopsy for PDA Diagnosis	Reference
cfDNA	cfDNA fragmentation pattern	plasma	Machine learning models integrating cfDNA fragmentation patterns, including fragment sizes and end motifs.	Yin et al. (2025) [30]
cfRNA/EV	13 miRNAs (5 cfRNA and 8 EV RNA)	plasma/serum	The combination of 13-miRNA signature archived an area under the curve (AUC) of 0.98 in training cohort and 0.93 in validation cohort for PDA detection.	Nakamura et al. (2022) [44]
EV	EV miRNA-based detection set	urine	Machine learning of urinary extracellular vesicle miRNA combinations showed high diagnostic accuracy for PDA, including early-stage disease.	Baba et al. (2024) [45]
EV	MUC5AC	plasma	MUC5AC in plasma EVs was shown to distinguish high-grade IPMNs from low-grade IPMNs with high sensitivity and specificity.	Yang et al. (2021) [48]
EV	lncRNA HEVEPA	serum	HEVEPA expression was upregulated in serum EVs from PDA patients compared to healthy controls and IPMN patients, achieving an AUC of 0.86.	Takahashi et al. (2024) [49]
protein	APO2-iTQ	blood	APOA2 isoform index (APO2-iTQ) has been implemented as an adjunctive marker for PDA diagnosis in Japan.	Hanada et al. (2024) [54]
protein	EphA2-NF	serum	EphA2-NF is associated with poor prognosis among patients receiving GnP treatment, and has potential clinical utility as both a diagnostic and prognostic marker.	Sato et al. (2023) [55]
cfDNA	methylated CpG tandem amplification	plasma	Methylation scoring and typing system achieved a sensitivity of 97% and 86% for patients in the discovery and validation cohorts, respectively, with a specificity of 100% in both cohorts for PDA.	Hu et al. (2025) [28]
cfDNA	methylated Homeobox A1 (mHOXA1) and methylated somatostatin (mSST)	serum	Analysis of mHOXA1 and mSST combination with CA19-9 showed to be useful to detect early stage of PDA.	Suehiro et al. (2022) [29]
cfDNA	KRAS	DF	KRAS mutation analysis of DF collected after secretin administration showed high diagnostic accuracy for PDA.	Yachida et al. (2025) [56]
protein	S100P	DF	The sensitivity and specificity of S100P protein expression in DF for diagnosing stages 0/IA/IB/IIA PDAC were 85% and 77%, respectively, with an AUC of 0.82.	Ideno et al. (2020) [58]
EV	circRNAs (circ_0006220 and circ_0001666)	plasma	circ_0006220 and circ_0001666) were found to correlate with CA19-9 levels, tumor size, and lymph node metastasis; the combination of these two circRNAs yielded an AUC of 0.884 for PDA diagnosis.	Hong et al. (2022) [59]
EV	10 miRNAs	plasma	Ten miRNAs highly expressed in the body fluids of patients with PDA was selected using public databases; These miRNAs were identified and verified as EV-miRNA candidates for early detection.	Makler et al. (2022) [60]
EV	4 miRNAs	plasma	EV-miRNA panel comprising four miRNAs (miR-93-5p, miR-339-3p, and miR-425-5p/3p) achieved diagnostic accuracy comparable to or greater than CA19-9 (AUC 0.885).	Makler et al. (2023) [61]
EV	molecular clustering of miRNAs	plasma	CT imaging features (radiomics) with the expression analysis of plasma EV-derived miRNAs (e.g., miR-1260b), improved the accuracy of differentiating malignant from benign pancreatic lesions to an AUC > 0.90.	Xu et al. (2025) [62]
EV	miRNAs (e.g., miR-21, miR-10b, miR-451a)	blood	Systematic review demonstrated the utility of plasma and serum EV-derived miRNAs (e.g., miR-21, miR-10b, miR-451a) in the diagnosis of PDA.	Patel et al. (2025) [63]
EV	HULC	serum	EV encapsulated HULC in serum was increased in serum derived from patients with PDA with AUC of 0.92.	Takahashi et al. (2020) [64]

DF, duodenal fluid.

## Data Availability

The original contributions presented in this study are included in the article. Further inquiries can be directed to the corresponding author.

## Abbreviations

The following abbreviations are used in this manuscript:

PDA	pancreatic ductal adenocarcinoma
cfDNA	cell-free DNA
cfRNA	cell-free RNA
EVs	extracellular vesicles
CTCs	circulating tumor cells
PanIN	pancreatic intraepithelial neoplasia
IPMN	intraductal papillary mucinous neoplasms
DF	duodenal fluid
PJ	pancreatic juice
CGP	cancer genome profiling
TAT	turnaround time
VAF	frequency
CHIP	clonal hematopoiesis of indeterminate potential
dPCR	digital PCR
ncRNAs	non-coding RNAs
miRNAs	microRNAs
lncRNAs	long non-coding RNAs
AUC	area under the curve
APO2-iTQ	APOA2 isoform index
GnP	gemcitabine plus nab-paclitaxel
AI	artificial intelligence
CNV	copy-number variation
MCTA-Seq	methylated CpG tandem amplification and sequencing
GPC1	Glypican-1
